# The systemic lupus erythematosus travel burden survey: baseline data among a South Carolina cohort

**DOI:** 10.1186/s13104-016-2060-0

**Published:** 2016-04-29

**Authors:** Edith M. Williams, Kasim Ortiz, Jiajia Zhang, Jie Zhou, Diane Kamen

**Affiliations:** Department of Public Health Sciences, Medical University of South Carolina, 135 Cannon Street, Suite 303, Charleston, SC USA; Department of Health Services Policy and Management, University of South Carolina, Columbia, SC USA; Department of Epidemiology and Biostatistics, University of South Carolina, Columbia, SC USA; Division of Rheumatology and Immunology, Department of Medicine, Medical University of South Carolina, Charleston, SC USA

**Keywords:** Systemic lupus erythematosus, Travel, Quality of life, Healthcare, Access

## Abstract

**Background:**

Many studies on the impact of systemic lupus erythematosus or lupus have identified patient travel costs as being problematic. We administered a survey that examined the impact of self-rated travel burden on lupus patients. The systemic lupus erythematosus travel burden survey included 41 patients enrolled in the systemic lupus erythematosus database project at the Medical University of South Carolina.

**Results:**

Most participants reported that travel caused medications to be discontinued or appointments to be missed. In unadjusted logistic regressions of the relationship between these outcomes and medical travel burden, both distance to rheumatologists and time to lupus medical care were significant.

**Conclusions:**

Our findings suggest that more research is needed to examine the influence of travel burden among this population, but data from this report could help to inform physicians, academic researchers, and other health professionals in South Carolina and other areas with significant rural populations on how travel burden may impact patients receiving care for lupus and provide an opportunity for the development of interventions aimed at assisting lupus patients with management of stressors related to travel burden.

## Background

Systemic lupus erythematosus (SLE) is a complicated disease to diagnose and treat, and the associated symptoms can be mentally and physically devastating for a patient [1, 2]. For many patients, these problems are magnified by the travel burdens they must endure. Travel burdens that SLE patients experience are largely due to the costs of driving to doctor’s visits, but this is multiplied by the fact that patients may have to visit a physician many times before being correctly diagnosed [3]. Additionally, once they have been correctly diagnosed, they will most likely need to see a specialist or multiple specialists [4]. In many cases, specialists are many miles away or the method of transportation that the patient has access to takes extensive amounts of time to get them there. Travel burden also encompasses barriers to care due to the negative impact that the disease can have on a SLE patient’s ability to access care [5]. Along with the distance and time it may take to get to a provider, travel burden may also include the pain associated with traveling to and from the doctor’s office and/or pharmacy locations [6, 7] as well as difficulty finding someone that will accompany them.

Disease impact of SLE has been studied extensively, including aspects of employment and the impact of SLE on medical and non-medical costs [8–12]. Many of these studies have identified patient travel costs as being problematic, eluding to negative impacts of social determinants of health. SLE patients are employed at a lower rate than the general population [13], which further exacerbates the problem. However, no studies have explicitly sought to examine ways to mediate the adverse influence of social determinants of heath on disease outcomes, especially regarding the concept of travel burden [8, 14–21]. The current study administered a validated survey and obtained baseline data to examine self-rated travel burden and characterize geographic accessibility of facilities most utilized by SLE patients in South Carolina.

## Methods

### Development and validation of the SLEOTB survey

The systemic lupus erythematosus observations of travel burden (SLEOTB) project developed a scale for measuring potential travel burden by conduct preliminary interviews to gain baseline knowledge of travel burden experienced by SLE patients in South Carolina, developing a survey instrument that to characterize observations, and pre-testing the survey through cognitive interviewing sessions. More detailed information on development of the survey tool-the background, literature search strategy applied, results of qualitative studies performed, and pre-testing for usability-is reported elsewhere [22]. For a community perspective on issues of travel burden (e.g., costs of travel, health care impacts of travel burden, visitation frequency for various health care needs, etc.), individual interviews were conducted with ten (10) SLE patients enrolled in the SLE Database Project at the Medical University of South Carolina (MUSC). Patients were randomly selected from the over 1000 SLE patients currently being followed at MUSC. A coding tree was developed to assist with coding of this data. Interviews were coded using NVivo software (Qualitative Solutions and Research, Pty Ltd, Victoria, Australia), a qualitative analysis research tool. Knowledge gained through the preliminary interviews assisted in determining which categories of analysis from the preliminary interviews still held valid for the construction of the survey.

Major focus areas of the initial survey instrument were: (1) travel patterns for health care; (2) perceptions of travel burden for health care; (3) perception of discrimination and its impact on healthcare utilization (pooled from two validated sources-reactions to race module from the behavioral risk factor surveillance system [23, 24] and Victoroff’s Oppression Questionnaire [25]); and (4) perceptions of how travel burden impacts disease management and seeking health. To ensure the validity of the survey instrument, cognitive interviews were conducted with 15 randomly selected participants from the MUSC SLE Database who did not participated in the preliminary interviews. Cognitive interviewing has been shown to improve survey questionnaires [26–30]. In order to further refine data on thematic issues relevant and specific to the types of self-rated travel burden that SLE patients in South Carolina experience across various socio-economic strata, the cognitive interviews were analyzed using the cultural consensus model [31–34]. The cultural consensus model is a recent innovation in ethnographic methods that has been shown to be sensitive to intra-cultural diversity as well as effects of stressors by social and cultural context [35]. Cultural consensus modeling was performed using Anthropac software (Columbia, Anakytic Technologies).

The resulting survey instrument assesses the impact that self-rated travel burden has on SLE patients regarding: (1) visitation frequency for primary care/rheumatologist/immunologist; (2) participation in clinical trials; (3) non-health related issues; and (4) how self-rated travel burden compares across urban/rural divides. The survey also includes questions concerning quality of life measures, costs for travel to health care, socio-demographic information, estimated time spent for travel to healthcare, and preferred mode of transport. Questions contained in the 55-item questionnaire are structured in a likert-scale manner, for measuring the strength of responses. Each component of the survey also provides space for open-ended response, allowing more in-depth responses and giving participants the opportunity to provide additional information they may deem relevant.

### Patients and entry criteria

Patients invited for survey administration were SLE patients attending rheumatology clinics at MUSC. All SLE patients met at least four components of the 1997 American College of Rheumatology (ACR) revised criteria for SLE [36], were 18 years of age or older, and were residing in South Carolina at the time of the study. Patients invited to participate in the proposed study were lupus patients participating in a longitudinal observational web-based SLE Database at MUSC. There were 402 patients with lupus enrolled in the Database during enrollment in this study. Patients in the Database were characterized longitudinally for disease activity and quality of life. As part of the informed consent process, participants agreed to future re-contact regarding other research studies. MUSC’s SLE cohort is geographically diverse, representing more than 60 South Carolina, Georgia, and North Carolina counties. Of the 402 patients with lupus, 336 were African–American. This study was approved by the University of South Carolina (USC) and MUSC Institutional Review Boards and written consent was obtained prior to data collection.

### Recruitment

For the current study, a link to a description of the study was placed on the MUSC lupus erythematosus (MUSCLE) group’s website and their listservs were used to email potential participants. Recruitment letters were also mailed and phone calls made to MUSC SLE database participants, and flyers were posted in corresponding lupus clinics. Survey administration was offered to participants in the most convenient format for them. This included telephone, online, or in-person administration. Patients were asked during recruitment which method they preferred. An online-version of the instrument was developed and made available on MUSC’s Research Electronic Data Capture (REDCap) system, a secure, web-based application designed exclusively to support data capture for research studies [37, 38]. Target enrollment for the survey was 148 participants. Although we were only able to secure roughly 28 % of participants from the original target, this relatively small sample should not have any impact on findings as SLE patients can be difficult to enroll in studies in general [39–41]. While the survey instrument obtained both qualitative and measures more readily handled with quantitative analyses, this paper focuses on quantitative analyses. For a review of qualitative work in this area, please see Ortiz, Flournoy-Floyd, & Williams, 2015 [42].

### Measures

The main variables assessed to examine health-related travel burden among SLE patients included travel time (in minutes) to lupus-associated medical care, and distance (in miles) to rheumatologists of lupus patients. To characterize travel burden more broadly, participants were asked to rate several domains in which travel could produce burden: (1) difficulty keeping appointments; (2) difficulty with general medical care travel; (3) difficulty with primary care travel (travel to/from primary care physicians); and (4) difficulty with rheumatologist travel (travel to/from rheumatologist). In distinguishing between travel for various aspects related to lupus patients care, it was our intent to be able to isolate specific travel burden for rheumatologist considering this subspecialty is vitally important for lupus patients. Moreover, being able to assess travel in the domains of general medical travel (e.g., travel for medications) and travel for primary care allows us to more thoroughly characterize travel burden. Response options for each of these measure was a likert scale (1, very difficult; 2, difficult; 3, neither; 4, easy; 5, very easy). All measures rely on self-reported data from patients participating in this survey. We utilized four outcome measures to characterize travel burden: (1) whether travel affected appointments (yes/no); (2) whether travel caused medications to be discontinued (yes/no), (3) whether medical transportation increased stress (yes/no), and (4) number of appointments missed due to transportation problems in the past year (count measure). Additionally, we control for several sociodemographic characteristics which have been shown to be impactful along the casual pathway in contributing to stress among lupus: (1) race (white, black); (2) age (in years); (3) gender (male/female); (4) educational attainment (equal to or less than high diploma or equivalent/college degree or higher); (5) employment status (yes/no); self-rated health status (good/fair); annual household income (<$15,000; $15,000–$60,000; ≥$60,000); marital status (married, never married, other).

### Data analysis

Input of data from telephone, online, and in-person surveys was completed throughout survey administration, and data was exported from the REDCap system in an excel format and manipulated using SAS statistical software. Survey data was analyzed utilizing statistical methods most appropriate for the sample size and descriptive statistics generated. First, we provide sociodemographic characteristics of participants. Then we describe travel burden by using measures to characterize various domains in which travel could produce burden by presenting descriptive statistics (Tables 2, 3, 4). For group comparisons of the interactions of travel burden and personal attitudes about travel burden, the Kruskal–Wallis test was applied. In order to investigate the association between travel burden and medical care, we focused on our four primary variables. For (1) travel affected appoints, (2) whether travel caused medications to be discontinued and (3) whether medical transportation increased stress we utilized logistic regression models. For the last outcome measure, number of appointments missed as a result of transportation problems in the past year, we utilized Poisson regression models. To further study the relationship between medical travel burden and selected outcomes (medication discontinuation and missed appointments) after adjusting for possible variables including education level, employment status, self-valuated health status, annual household income and marital status, multivariable logistic or Poisson regression models were constructed.

## Results

A total of 41 patients participated, and 39 completed the survey, corresponding to an approximate 28 % response rate. Table 1 summarizes demographic characteristics of participants. Among all participants, the average age is 43.15 (range 23–75), 89.2 % are female, 65.0 % are African American, 76.3 % patients had an education level of college or higher; about half (53.9 %) of participants were currently employed; 70 % perceive their health status as ‘good’; about half (48.7 %) of the patients had an annual household income between $15,000 and $60,000; and about half (51.7 %) of survey participants were married. Most patients (92.3 %) currently had health insurance. About a quarter of participants (25.7 %) reported Medicaid as their primary insurance provider, and 26.9 % had Medicare as their primary insurance provider. Only 23.1 % of participants reported that their insurance covered medical transportation. Table 2 shows that the mean travel time to lupus-associated medical care was approximately 57 min (ranging from 4 to 150 min), and the average distance to rheumatologists was approximately 53 miles (ranging from 4 to 200 miles).

**Table 1:** Demographic characteristics of SLE travel burden survey participants (N = 39)

Gender	
Female	33 (84.62 %)
Male	4 (10.26 %)
Missing	2 (5.12 %)
Race	
White	13 (32.5 %)
Black, or African–American	26 (65.0 %)
Age	
Mean (SD)	43.15 (12.92)
Median (Q1–Q3)	41.5 (32, 49.25)
Min–max	(23, 75)
Education level	
≤High school	9 (23.08 %)
≥College	29 (74.36 %)
Missing	1 (2.56)
Employment status	
Employed	21 (53.85 %)
Other	18 (46.15 %)
Self-evaluated health status	
Good	21 (53.85 %)
Fair	9 (23.08 %)
Missing	9 (23.08 %)
Annual household income	
<$15,000	9 (23.08 %)
Between	19 (48.72 %)
>$60,000	11 (28.21 %)
Marital status	
Married	15 (38.46 %)
Never married	6 (15.38 %)
Other	8 (20.51 %)
Missing	10 (25.64)
Currently have health insurance	
Yes	36 (92.31 %)
No	3 (7.69 %)
Primary insurance medicaid	
Yes	9 (23.08 %)
No	26 (66.67 %)
Missing	4 (10.26 %)
Primary insurance medicare	
Yes	7 (17.95 %)
No	19 (48.72 %)
Missing	13 (33.33 %)
Insurance covering medical transportation	
Yes	9 (23.08 %)
No	30 (76.92 %)

Self-rated health status was assessed with the following question: in general, what would you say your health is….? [48]

**Table 2:** Health-related travel burden of SLE travel burden survey participants (N = 39)

Distance to rheumatologist (miles)	
Mean (Std)	52.94 (47.04)
Median (Q1–Q3)	40 (10–78)
Min–max	4–200
Travel time to Lupus medical care (minutes)	
Mean (Std)	57.01 (41.67)
Median (Q1–Q3)	45 (20–90)
Min–max	4–150

Table 3 describes participants’ attitudes toward medical care travel. 30.6 % of participants considered it easy to keep medical appointments, compared with 33.3 % of participants who reported keeping appointments as difficult. 42.5 % of participants described their travel for medical care as easy, compared with 37.5 % who reported it as difficult. Regarding rheumatologist travel, 42.5 % of participants felt travel to their rheumatologist was easy, while 40 % of participants found it difficult. This trend was more pronounced with respect to primary care travel. 62.5 % of participants felt travel for primary care purposes was easy, while 20 % of participants found it difficult.

**Table 3:** SLE travel burden survey attitude toward medical care travel (N = 41)

Do travel issues make keeping your appointments	
Difficult	12 (30.77 %)
Neither	13 (33.33 %)
Easy	11 (28.21 %)
Missing	5 (12.20 %)
How would you describe travel for medical care travel	
Difficult	15 (36.59 %)
Neither	8 (19.51 %)
Easy	17 (43.59 %)
Missing	1 (2.44 %)
How difficult is it for you to travel to your primary care provider	
Difficult	8 (19.51 %)
Neither	7 (17.07 %)
Easy	25 (60.98 %)
Missing	1 (2.44 %)
How difficult is it for you to travel to your rheumatologist	
Difficult	16 (39.02 %)
Neither	7 (17.07 %)
Easy	17 (43.59 %)
Missing	1 (2.44 %)

Table 4 shows that 35.9 % of participants felt that travel affected whether they were able to keep appointments, 12.8 % of participants reported discontinuing medications as a result of travel issues, and 66.7 % reported feeling more stressed as a result of medical care transportation. Number of medical appointments missed during the last year due to transportation issues ranged from 0 to 5 appointments across all participants, but more than half of them (55.3 %) had not missed any appointments.

**Table 4:** SLE travel burden survey primary outcome variables (N = 39)

Do you feel travel affects whether you are able to keep your appointments	
Yes	14 (35.90 %)
No	25 (64.10 %)
Have travel issues ever resulted in your medications being discontinued as a result of missed appointments	
Yes	5 (12.82 %)
No	34 (87.18 %)
Would you agree that transportation issues for medical care increases stress	
Yes	26 (66.67 %)
No	13 (33.33 %)
In 1 year, how many appointments have you missed/rescheduled because of transportation problems	
0	21 (53.85 %)
1–2	6 (15.38 %)
3–5	11 (28.21 %)
Missing	1 (2.56 %)

**Fig. 1:**
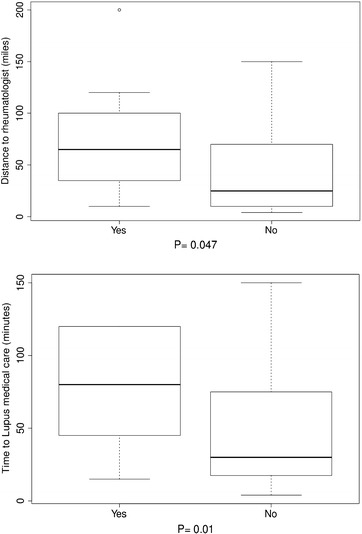
Travel affects appointments

**Fig. 2:**
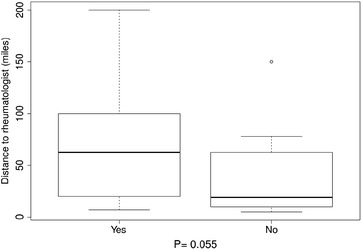
Medical care transportation increases stress

**Fig. 3:**
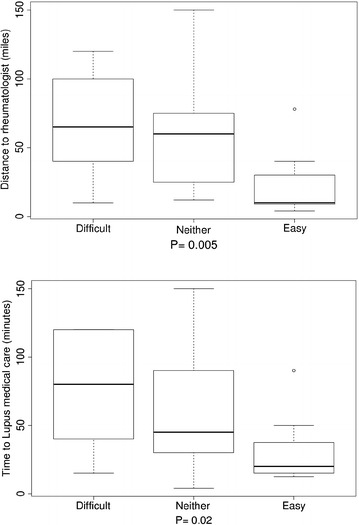
Difficulty for keeping appointments

**Fig. 4:**
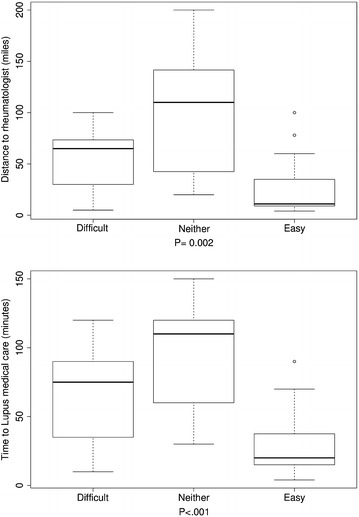
Difficulty for medical care travel

**Fig. 5:**
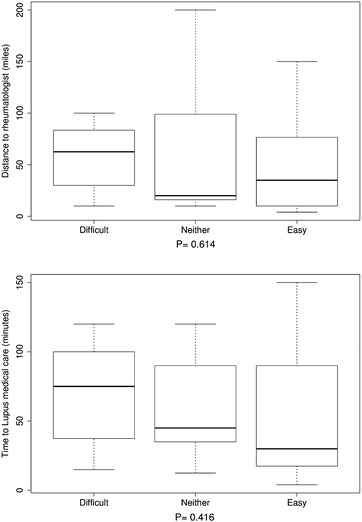
Difficulty for primary care travel

Further investigation of the interactions of travel burden and personal attitudes toward travel burden revealed that the distance to rheumatologists, for patients who reported that travel affected their appointments, was significantly longer (P 0.047 in Fig. 1a) and they reported significantly longer time to lupus-associated medical care (P 0.01 in Fig. 1b) compared with those who reported that travel did not affect their appointments. Those who reported that medical care transportation increased stress had longer distances to their rheumatologists compared with patients who reported that medical care transportation did not cause them more stress, but this finding did not reach statistical significance (P 0.055 in Fig. 2). There were significant differences (P < 0.05) in the distance to rheumatologists and time to lupus medical care for those participants who reported difficulties in keeping medical appointments as a result of travel burdens, compared with those who reported no difficulty. Usually, participants reporting difficulty keeping medical appointments had longer distances to their rheumatologists and spent more time getting to lupus medical care (Fig. 3a, b). Similarly, for those participants who noted difficulties with medical care travel, significant differences (P < 0.01) existed in the distance to rheumatologists and time to lupus-associated medical care. Again, most participants reporting difficulty with medical care travel had longer distances to their rheumatologists and spent more time getting to lupus-associated medical care (Fig. 4a, b). There were no significant differences (P > 0.05), for participants who reported difficulties with primary care travel, in the distance to rheumatologists and time to lupus-associated medical care (Fig. 5a, b). For those participants who reported difficulties with travel to their rheumatologist, significant differences (P < 0.05) existed in the distance to rheumatologists and time to lupus-associated medical care. Both distance to rheumatologists and time to lupus-associated medical care were significant at level 0.1 in unadjusted regression models with outcomes of whether travel affected appointments and number of appointments missed due to transportation issues, but they became insignificant after adjustment for health status, income level, and car ownership. Both distance and time to medical care were not significant with outcomes of medication discontinuation and transportation issues increasing stress, in both unadjusted and adjusted logistic regression models. (see Table 5).

**Table 5:** Unadjusted and adjusted models of the effects of travel on appointments

Outcome: travel affects appointments	Unadjusted model			Adjusted model		
Predictor	Estimate	Std. error	P value	Estimate	Std. error	P value
Time to lupus medical care	0.022	0.010	0.019*	0.218	0.239	0.36
Distance to rheumatologists	0.015	0.008	0.069*	0.031	0.035	0.368
Outcome: travel discontinued medications						
Time to lupus medical care	0.013	0.011	0.269	−0.003	0.023	0.908
Distance to rheumatologists	0.004	0.010	0.647	−0.009	0.023	0.68
Outcome: medical care transportation increases stress						
Time to lupus medical care	0.010	0.009	0.263	0.354	0.337	0.293
Distance to rheumatologists	0.014	0.009	0.147	0.061	0.067	0.362
Outcome: number of appointments missed due to transportation						
Time to lupus medical care	0.008	0.003	0.014*	0.006	0.014	0.635
Distance to rheumatologists	0.005	0.003	0.070*	0.001	0.01	0.958

*Adjusted model* adjusted for age, gender, education level, employment status, self-evaluated health status, annual household income, and marital status

* Level of significance P < 0.05 for each category

## Discussion

Further study could include utilizing GIS techniques to characterize such travel burden by comparing self-rated travel burden (e.g., travel distance for healthcare, travel costs for healthcare) with actual travel burden (e.g., utilizing place of residence and facility address to map travel distances). These techniques are valuable in understanding the expanded scope of barriers to include SLE health-related travel [43]. Taxi-cab distance measures could be calculated in ArcMap to provide travel times.

There is also a critical gap in our understanding of urban/rural differences in travel burden among SLE patients. Due to incomplete responses, we were unable to present and analyze information on urban vs. rural residence. However, South Carolina provides the ideal environment for study and future inquiries are planned. Utilizing the MUSC SLE Database, which includes patients across the state of South Carolina, provides a unique opportunity to characterize urban/rural issues that may not be permitted in other cohort studies that are mainly organized in urban areas. While it is recognized that interviewees may not be nationally representative, this population could provide us with knowledge pertaining to South Carolina and its uniqueness in urban/rural divisions, and lead to broader application to other areas with significant rural populations. Interestingly a majority of respondents had health insurance, which greatly varies from nationally representative studies among SLE patients which demonstrates that many SLE patients have public insurance (e.g., Medicaid) [44]. Although it should be noted that insurance rates in our sample (roughly 25 % Medicaid beneficiaries, and roughly 26 % of participants were Medicare beneficiaries) were comparable to state analyses of SLE patients (roughly 55 % SLE patients had public insurance) [45]. Most of the participants identified that their public insurance had associated support for medical transportation, which varies greatly compared to most persons nationally [46].

Other limitations of the current study include limited information provided on the nature of the sample, particularly SLE disease characteristics and a small sample size limiting the ability to perform significant modelling or data exploration. It is crucial that other groups collaborate in validating the survey instrument in external cohorts to confirm and further support our findings and conclusions. Future directions could include characterization of the role of residence and how observed trends may vary buy rurality/urbanity. In one study, rural residents on average traveled eight miles farther than urban residents. The study further states that it also took African–Americans more time to get to their provider compared to their white counterparts [47] but it does not specifically address individuals with SLE and the type of specialist care that is necessary in that context.

## Conclusions

Our findings indicate that medical transportation increased stress for most participants. Travel burden pertaining to distance and time shows that longer distance and time are associated with negative outcomes like increased pressure on medical appointments and causing medications to be discontinued or appointments to be missed. Travel burden needs to be studied extensively with the understanding that the burden is based on the perspective of the individual. Particularly, this data could inform development of interventions aimed at assisting SLE patients with management of stressors related to travel. This project will also provide an opportunity for physicians, academic researchers, and other health professionals in South Carolina and other areas with significant rural populations to gain a better understanding of how travel burden may impact patients receiving care for SLE, while also informing much broader contexts of how to improve participation of SLE patients in future studies.
